# Obesity Simulating Pregnancy—Caution in the Diagnosis of Pregnancy

**Published:** 1850-04

**Authors:** Leopold Schonburgh


*Obesity Simulating Pregnancy—Caution in the Diagnosis of Pregnancy.* By Dr. Leopold Schonburgh.—Mrs. ——, whose husband had been separated from her by imprisonment upwards of three months, after exposure to cold, experienced all the symptoms of pregnancy. She was a modest and virtuous woman, and believed herself to have been three months pregnant. In about eight weeks more she believed she felt the movements of the child; the abdomen continued to increase in size proportionably. At the end of nine months her abdomen presented the appearance of the full period of gestation, but she had not felt the supposed fœtal movements for several weeks. The catamenia now returned regularly at each month, her health was good, and the size of the abdomen again decreased to the size of about five months' pregnancy.

The umbilicus was depressed, the parietes felt doughy, free from fluctuation, the hands could be pressed below them four or five inches downwards towards the spine, and could be made to meet together beneath the fat integuments; the uterus could be felt somewhat enlarged in the hypogastric region. On examination per vaginam, the os uteri could be readily reached: it was soft and seemed swollen; two lateral cicatrices could be perceived on its surface. The cervix uteri was rather more than half an inch in length. The posterior wall was soft and rather tender; pressure on the abdomen could be felt to depress this organ. The mucous membrane of the vagina did not present a bluish, but the ordinary red colour.

It was agreed that no pregnancy existed in this case, but that the suppression of the catemenia by cold had given rise to the rapid development of fat, and congestion of the pelvic viscera, with the consequent enlargement of the abdomen.—*Casper's Wochenschrift and Monthly Jour.*

